# Enrichment of Flavonoids in Short-Germinated Black Soybeans (*Glycine max* L.) Induced by Slight Acid Treatment

**DOI:** 10.3390/foods13060868

**Published:** 2024-03-13

**Authors:** Caimei Huang, Xiaolan Quan, Yongqi Yin, Xiangli Ding, Zhengfei Yang, Jiangyu Zhu, Weiming Fang

**Affiliations:** 1 College of Food Science and Engineering, Yangzhou University, Yangzhou 225009, China; 212403104@stu.yzu.edu.cn (C.H.); mz120211954@stu.yzu.edu.cn (X.Q.); yzf@yzu.edu.cn (Z.Y.); 008051@yzu.edu.cn (J.Z.); wmfang@yzu.edu.cn (W.F.); 2 School of Tourism and Cuisine, Yangzhou University, Yangzhou 225009, China; dingxl@yzu.edu.cn

**Keywords:** flavonoid, black soybean, short germinated, slight acid treatment, response surface methodology

## Abstract

Exogenous abiotic stimulant treatments are a straightforward and effective method for enhancing secondary metabolites in plants. In this study, the response surface optimization method was used to optimize the conditions for enriching flavonoids in short-germinated black soybeans under a slight acid treatment, and the mechanism of flavonoid accumulation during black soybean germination was explored. The results show that the use of a 126.2 mM citric acid–sodium citrate buffer (pH 5.10) as a slight acid treatment resulted in the highest flavonoid content when the black soybeans were germinated for 24 h. Under these conditions, the isoflavonoid (glycitin, daidzein, and genistein) increased significantly, and the flavonoid content reached 2.32 mg/g FW. The microacidified germination treatment significantly increased the activities and relative gene expression levels of key enzymes involved in flavonoid metabolism (4-coumarate-CoA ligase and cinnamic acid 4-hydroxylase, etc.). However, the slight acid treatment inhibited the growth of the black soybeans and caused damage to their cells. This was evidenced by significantly higher levels of malondialdehyde, superoxide anion, and hydrogen peroxide compared to the control group. Furthermore, the antioxidant system in the short-germinated soybeans was activated by the slight acid treatment, leading to a significant increase in the activities and relative gene expression levels of catalase and peroxidase. The results above show that a slight acid treatment was beneficial in inducing the accumulation of flavonoids during the growth of black soybean sprouts. This lays a technical foundation for producing black soybean products that are rich in flavonoids.

## 1. Introduction

Flavonoids are bioactive compounds found in a variety of soybeans, primarily consisting of daidzin, daidzein, glycitin, genistein, genistin, and genistein. Flavonoids are structurally like mammalian estrogen. When consumed in high amounts, they can have beneficial effects on human health [1], such as preventing and treating osteoporosis [2], alleviating postmenopausal symptoms in women [3], and inhibiting the growth of cancer cells by affecting genes related to the cell cycle and apoptosis [4]. Therefore, enhancing the flavonoid content in soybeans has become a research focus. Research has demonstrated that germination is a simple and cost-effective method for enhancing the bioactive substances of soybeans [5]. In particular, the content of isoflavones, such as daidzein, genistein, and daidzein, can be significantly increased during soybean germination [6].

Soybeans (*Glycine max* L.) are versatile and nutritious legumes with high levels of bioactive compounds, such as isoflavones [7], saponins [8], anthocyanins [9], tocopherols [10], and more. These compounds have garnered global attention as functional dietary ingredients. Desta et al. [11] demonstrated that the composition and concentration of phytochemicals in soybeans vary significantly based on the seed coat colors. Black soybeans exhibit higher antioxidant activity due to their elevated levels of isoflavones [12]. Many plant-based foods also reduce anti-nutrient factors, such as phytic acid, tannins, and α-galacto-oligosaccharides, and increase bioactive substances, especially flavonoids, through short-term germination. The study by Shi et al. [13] found that germinated wheat soy sauce contained higher levels of isoflavones and gamma-aminobutyric acid compared to unsprouted wheat. Germination reduces antinutritional factors in chickpea flour and enhances the nutritional and functional properties of chickpea flour [14]. However, there are few studies on enriching isoflavones in black soybeans through germination and using them as raw materials for developing functional foods.

Enzyme activities and gene expressions related to flavonoid metabolism in plants are regulated by developmental signals and can be readily induced by various external stimuli. When legumes are exposed to abiotic stress during germination, the isoflavone content is further increased. According to a study by Ma et al. [15], the isoflavone content in germinating soybeans increased as key enzyme activities in isoflavone biosynthesis were upregulated under UV-B illumination. The isoflavone content in germinating soybeans increased by up to 4.14 times after being treated with melatonin. pH also has a significant impact on both plant growth and the synthesis of its secondary metabolites. Acidification treatment significantly increases the accumulation of γ-aminobutyric acid during germination in soybean [16], brown rice [17], foxtail millet [18], and buckwheat [19]. Acidification treatment also induces the production of secondary metabolites in various plant cell suspensions by regulating key enzymes and their gene expression [20,21]. However, to the best of our knowledge, only a limited number of studies have investigated the effects of acid treatment on flavonoid biosynthesis and plant growth, particularly in germinated black beans.

The response surface methodology (RSM) was used in this study to optimize the parameters for enhancing flavonoids (germination time, pH, and concentration). This study investigated the physiological and biochemical changes in black soybean sprouts under a slight acid treatment and examined the correlation between these changes and flavonoid levels. The enzymatic activity and gene expression related to the antioxidant system and the phenylpropane pathway were analyzed to understand the regulatory mechanism of the slight acid treatment on flavonoid synthesis in short-germinated black soybeans. This study is helpful for understanding the method and mechanism of abiotic stress that promotes flavonoid accumulation in black soybeans during short-term germination. It lays a foundation for producing flavonoid-rich supplements and healthy foods.

## 2. Materials and Methods

### 2.1. Germination Process

The black soybean seeds (*Glycine max* (L.) Merr. cultivar Shengxing No. 1) were purchased from Zhangjiakou, Hebei Province. Seeds were sterilized with 1% sodium hypochlorite for 15 min, then rinsed with distilled water to remove residual chlorine until they reached a neutral pH. Subsequently, the seeds were immersed in distilled water for 6 h at 30 °C and then placed in a germination machine and incubated at 30 °C. During germination, the control group was sprayed with 30 mL of distilled water, while the experimental group was sprayed with a citric acid–sodium citrate buffer (126.2 mM, pH 5.10) every 12 h, respectively. Subsequently, about 0.5 g of black soybean sprouts were randomly sampled at specific germination times (0 h, 12 h, and 24 h) for analysis.

### 2.2. Optimizing Germination Conditions by Response Surface Methodology

After determining the initial range of the germination time, pH values, and treatment solution concentrations through single-factor tests (the data are shown in Supplementary Figure S1), a Box–Behnken design with three dependent variables was employed in this study. The settings for the independent variables were as follows (with low and high values): a germination time of 12 and 36 h, a pH of 4 and 6, and a treatment solution concentration of 90 and 150 mM. The range of independent variables and their levels are presented in Table 1. The Design-Expert software 13 (State-Ease Inc., Minneapolis, MN, USA) was utilized to analyze the experimental setup and calculate the anticipated data to estimate the response of the independent variables.

### 2.3. Determination of Total Flavonoid Content

The total flavonoid was extracted and determined according to the method of Vélez et al. [6]. Black soybean sprouts were ground in 5 mL of 80% (*v*/*v*) ethanol and sonicated at 30 °C for 25 min. After centrifugation at 9000× *g* for 10 min, the supernatant was recovered. The absorbance was measured at 260 nm, and genistein was used to produce the standard curve.

### 2.4. Determination of the Monomer Contents of Flavonoids

The contents of genistein, daidzein, glycitein, daidzin, and genistin were determined using the method described by Yin et al. [22]. Briefly, short-germinated black soybeans were ground in 80% methanol, and the homogenate was centrifuged at 12,000× *g* for 10 min to collect the supernatant. An Agilent 1200 HPLC system (Agilent Technologies Co. Ltd., Santa Clara, CA, USA) was used to analyze the extracted sample. The separation of the sample was achieved using a ZORBAX SB-C18 column (5 μm particle size, 4.6 × 250 mm, Agilent Technology Co., Ltd., Santa Clara, CA, USA). The HPLC parameters included solvent A (water with 0.1% acetic acid); solvent B (acetonitrile with 0.1% acetic acid); gradient elution, whereby the ratio of solvent B was increased (0 min–3 min–5 min–7 min–9 min–20 min–22 min–25 min–30 min, 10%–15%–30%–38%–43%–75%–80%–75%–10%); a detection wavelength of 260 nm; a flow rate of 0.8 mL/min; and a column temperature of 35 °C.

### 2.5. Determination of Malondialdehyde, Superoxide Anion, and Hydrogen Peroxide

The content of malondialdehyde (MDA) was determined following the protocol by Zhuang et al. [22] with slight modifications. More precisely, 0.5 g of black soybean sprouts were ground with 5.0 mL of 5% trichloroacetic acid, centrifuged at 8000× *g* for 10 min. A total of 2.0 mL of 0.76% thibabituric acid was added to the supernatant, mixed, and bathed for 30 min. The absorbance of the supernatant was measured at 450 nm, 532 nm, and 600 nm.

The contents of superoxide anion ($O_{2}^{-.}$) and hydrogen peroxide (H_2_O_2_) were measured according to Zhao et al. [23]. Take 0.5 g of black soybean sprouts, grind them with 65 mM of a phosphate buffer, and centrifuge at 8000× *g* for 10 min. Take 1 mL of the supernatant and mix it with 0.9 mL of 65 mM of the phosphate buffer (pH 7.8) and 0.1 mL of 10 mM of hydrochloric acid light amine. After incubating at 25 °C for 20 min, add 17 mM of *p*-aminobenzenesulfonic acid and 7 mM of *a*-naphthylamine to the mixture, and then measure absorbance at 530 nm after incubating for 20 min at 25 °C. Calculate the content of $O_{2}^{-.}$ in the sample based on the NaNO_2_ standard curve. Approximately 0.5 g of black soybean sprouts were taken, ground with 5 mL of 0.1% trichloroacetic acid, and centrifuged at 8000× *g* for 10 min. To 0.5 mL of the supernatant, sequentially add 0.5 mL of 0.1% trichloroacetic acid, 10 mM of the phosphate buffer (pH 7.0), and 1 mL of potassium iodide, shake well, and then incubate at 28 °C for 1 h, followed by measuring the absorbance at 390 nm.

### 2.6. Determination of Free Amino Acids and Soluble Proteins

The contents of free amino acids and soluble proteins were performed according to Yin et al. [22]. A total of 0.5 g of fresh sprouts were ground with 5.0 mL of 10% acetic acid and centrifuged at 8000× *g* for 10 min. The supernatant was heated at 100 °C for 15 min and rapidly cooled in an ice bath. The absorbance of the supernatant was measured at 570 nm. The soluble protein content in the black soybean sprouts was determined using the Bradford method. A total of 0.5 g of black soybean sprouts was ground with 5 mL of water and centrifuged at 8000× *g* for 10 min, and 0.1 mL of the supernatant was mixed with 0.9 mL of water and 5 mL of the Bradford solution. After 2 min, the absorbance was measured at 595 nm. A standard curve was prepared using bovine serum albumin.

### 2.7. Determination of Antioxidant Enzyme Activity

To measure the activities of ascorbate peroxidase (APX), superoxide dismutase (SOD), catalase (CAT), and peroxidase (POD), the sprouts were ground with a sodium–phosphate buffer (pH 7.0, 50 mM). Then, the solution was centrifuged at 12,000× *g* for 15 min at 4 °C. The SOD and APX activities were determined following the method of Yin et al. [24], where one unit of SOD activity corresponded to a change of 1 per min in OD*_560nm_* and OD*_290nm_*, respectively. The CAT and POD activities were measured as described by Yin et al. [22], with one unit of CAT and POD activity defined as a change of 0.01 per min in OD*_240nm_* and OD*_470nm_*, respectively.

### 2.8. Determination of Isoflavone Synthetase Activity

For the determination of phenylalanine ammonia lyase (PAL), cinnamic acid 4-hydroxylase (C4H), and 4-coumarate coenzyme A ligase (4CL) activities, the sprouts were ground with a Tris-HCl buffer (pH 8.9, 0.1 M). Then, the solution was centrifuged at 12,000× *g* for 15 min at 4 °C. The activities were determined according to the method described by Yin et al. [22], with one unit of PAL, C4H, and 4CL activity defined as a change of 0.01 per min in OD*_290nm_*, OD*_340nm_*, and OD*_333nm_*, respectively.

### 2.9. RNA Extraction and Quantitative Real-Time PCR Analysis

The total RNA was extracted from the short-germinated black beans using an E.A.N.A.^TM^ Plant RNA kit (R6827-01, OMEGA, Norcross, GA, USA). The RNA samples were reverse transcribed into cDNA using a PrimeScript™ RT Master Mix kit (RR036A, Takara, Japan). The quantification of each cDNA was performed in triplicate using SYBRR premix EX-Taq™ (RR420A, Takara, Japan) and a Light Cycler 480II detection system (Roche, Basel, Switzerland). The oligonucleotide primers used for qRT-PCR are listed in Supplementary Table S1. The relative gene expression levels were calculated using the 2^−ΔΔCt^ method.

### 2.10. Statistical Analysis

The experimental data were expressed as a mean ± standard deviation based on three replications. A statistical analysis was performed using one-way ANOVA and Tukey’s multiple tests, with a significance level of *p* < 0.05 and *p* < 0.01. A correlation analysis was conducted using the Origin 2021 software and the Pearson method.

## 3. Results

### 3.1. Optimization of Microacidified Germination Conditions for Flavonoid Production in Short-Germinated Black Soybeans

According to the results of a single-factor test, the most favorable conditions for the accumulation of flavonoids in black soybeans were a germination time of 24 h, a pH of 5.0, and a concentration of 120 mM, respectively (Supplementary Figure S1). According to the results of a single-factor experiment, a 17-run Box–Behnken design with three factors and three levels was utilized to create a second-order response surface and optimize the germination conditions. The results of the significance test and analysis of variance for the quadratic polynomial model are presented in Supplementary Table S2. The fit of the predictive model was assessed by determining the coefficient R^2^, which was 0.9987. The F-value for the lack of fit was not significant (*p* > 0.05), confirming the validity of the model. As indicated in Supplementary Table S2, the germination time, pH, and concentration had significant effects on the enrichment of flavonoids (*p* < 0.01). In addition, the interaction terms between the germination time and pH (*p* < 0.01) and between the germination time and concentration (*p* < 0.01) were important model factors for flavonoid enrichment. All the secondary factors, including the germination time, pH, and concentration, were found to be significant (*p* < 0.01). The parameters of the following Equation (1) were obtained through a multiple regression analysis of the experimental data:

Y = 2.30 + 0.0163X_1_ + 0.0700X_2_ + 0.0637X_3_ − 0.0225 X_1_ X_2_ + 0.0250 X_1_ X_3_ + 0.0125 X_2_ X_3_ − 0.2850 X_1_^2^ − 2575 X_2_^2^ − 0.1650X_3_^2^ (1)

**Figure 1:**
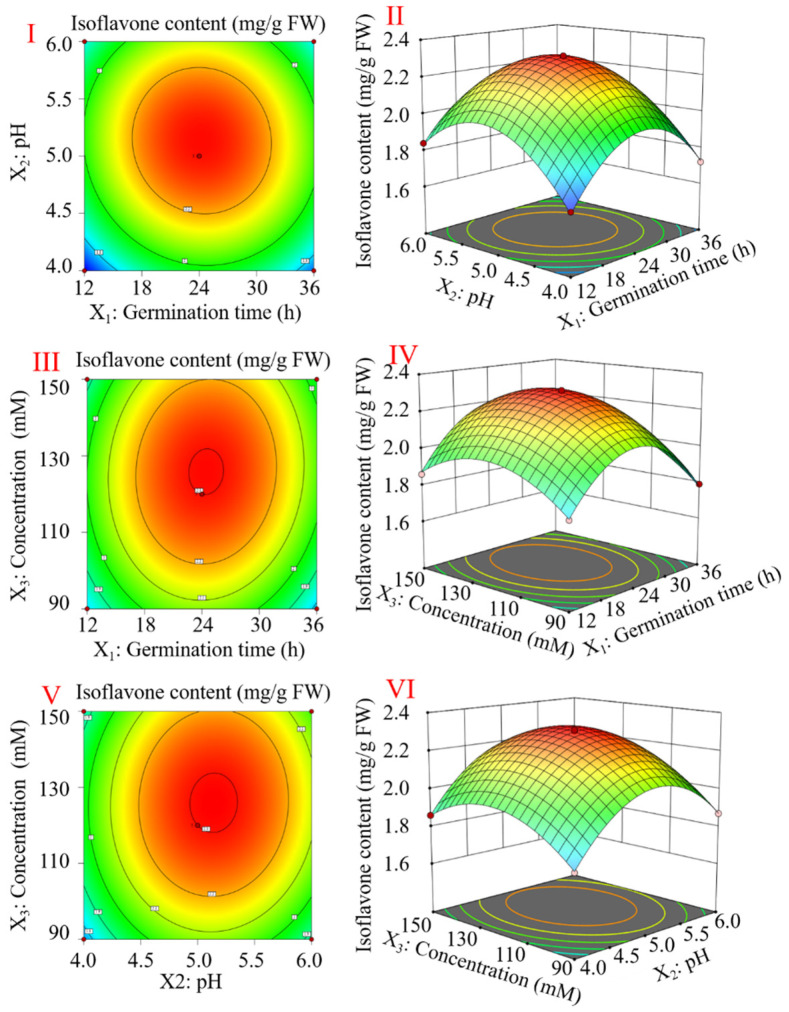
Three-dimensional plots and contour plots of the interaction of variables on flavonoid enrichment. The interaction between the germination time and pH of treatment solutions (**I**,**II**). The interaction between the germination time and concentration of the treatment solution (**III**,**IV**). The interaction between the pH of the treatment solution and the concentration of the treatment solution (**V**,**VI**).

Moreover, Figure 1 displays response surface plots and contour plots. Based on Figure 1 and Equation (1), the optimal germination condition for enhancing the flavonoid content involved spraying with a 126.2 mM citric acid–sodium citrate solution (pH 5.10) for 24 h. This condition resulted in the highest flavonoid content of 2.32 mg/g FW.

### 3.2. The Impact of Slight Acid Treatment on Flavonoid and Monomer Contents in Black Soybean Sprouts during Germination

**Figure 2:**
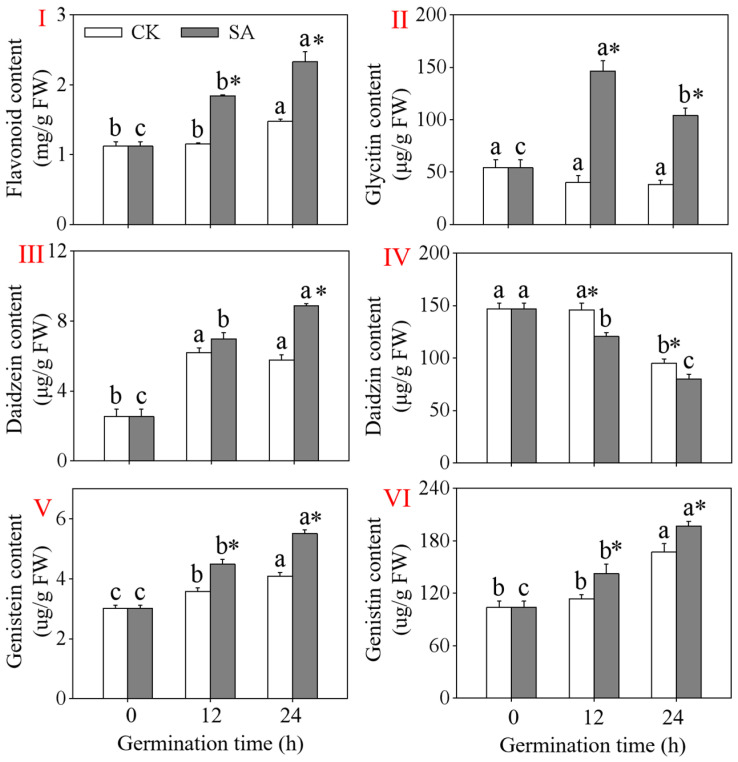
Effects of a slight acid treatment on the content of flavonoids (**I**), glycitin (**II**), daidzein (**III**), daidzin (**IV**), genistein (**V**), and genistin (**VI**) in short-germinated black soybeans. Different lowercase letters indicate significant differences among the germination time under the same treatment (*p* < 0.05). * Indicates a significant difference between the SA treatments and the CK at the same germination time (*p* < 0.05). SA: slight acid treatment. CK: water treatment.

A significant increase (*p* < 0.05) in the flavonoid content was observed in the short-germinated black soybeans under a slight acid treatment during germination (Figure 2I). The flavonoid content reached 2.32 mg/g FW after 24 h of germination, which was 1.53 times higher than that of the control (CK). Compared to the CK, the levels of genistin, daidzein, and genistein significantly increased after 24 h of the slight acid treatment in 24 h black soybean sprouts (Figure 2III,V,VI). The fluctuation in the daidzein, genistein, and genistin contents mirrors that of the flavonoid content. However, the daidzin content decreased as the germination time increased, which contradicted the trend in the flavonoid content. The glycitin content in the black soybean sprouts did not change significantly under the CK but increased initially and then decreased under the slight acid treatment (Figure 2II).

### 3.3. Effects of Slight Acid Treatment on Growth Performance and Physiological and Biochemical Indices of Black Soybean Sprouts

**Figure 3:**
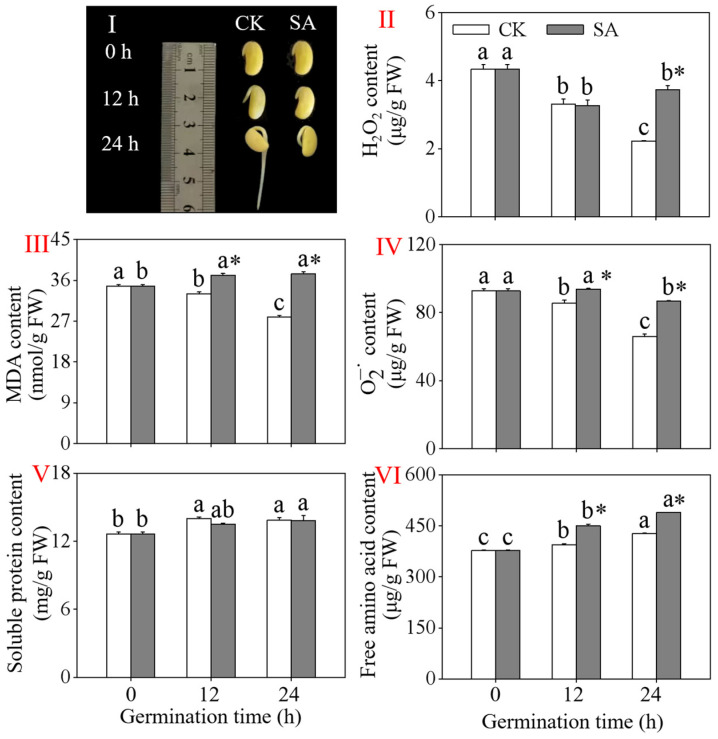
Effects of a slight acid treatment on the growth performance (**I**), H_2_O_2_ levels (**II**), MDA levels (**III**), $O_{2}^{-.}$ levels (**IV**), soluble protein content (**V**), and free amino acid levels (**VI**) of black soybean sprouts. Different lowercase letters indicate significant differences among the germination time under the same treatment (*p* < 0.05). * Indicates a significant difference between the SA treatments and CK at the same germination time (*p* < 0.05). SA: slight acid treatment. CK: water treatment.

The growth of the black soybeans was significantly hindered by the slight acid treatment, and the inhibitory effect on growth became more pronounced as the germination time increased (Figure 3I). As shown in Figure 3II–IV, the levels of H_2_O_2_, MDA, and $O_{2}^{-.}$ decreased significantly during germination under the CK treatment, with reductions of 48.62%, 19.86%, and 28.92% at 24 h compared to 0 h, respectively. At 24 h after the slight acid treatment, the MDA level dramatically increased, while the content of H_2_O_2_ significantly decreased (*p* < 0.05). Furthermore, the levels of MDA and H_2_O_2_ in the 24 h black soybean sprouts treated with slight acid were 1.36, 1.31, and 1.67 times higher than those of the control group, respectively (*p* < 0.05). The levels of free amino acids and soluble proteins increased with longer germination times under the CK and slight acid treatment (*p* < 0.05). These findings indicate that germination breaks the dormancy of seeds, while acid treatment delays the growth of black soybean sprouts.

### 3.4. Changes in Flavonoid Synthase and Antioxidase Activities of Slight Acid-Treated Black Soybeans during Germination

**Figure 4:**
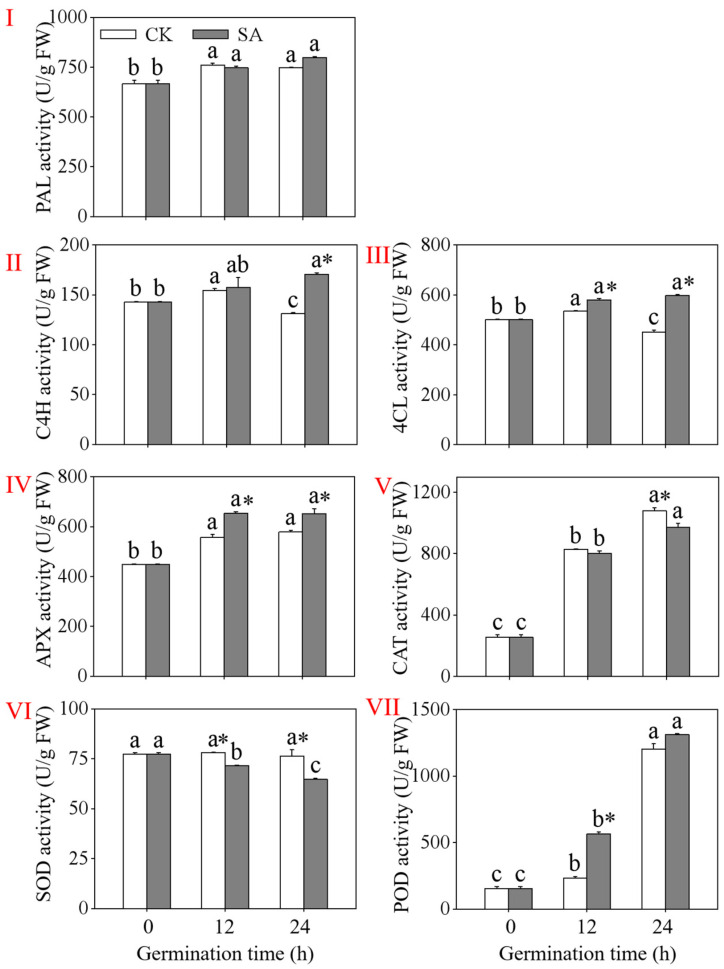
Effects of the slight acid treatment of black soybeans during germination on the activity of PAL (**I**), C4H (**II**), 4CL (**III**), APX (**IV**), CAT (**V**), SOD (**VI**), and POD (**VII**). Different lowercase letters indicate significant differences among the germination time under the same treatment (*p* < 0.05). * Indicates a significant difference between the SA treatments and CK at the same germination time (*p* < 0.05). SA: slight acid treatment. CK: water treatment.

During black bean germination, three key enzymes involved in flavonoid biosynthesis in plants, PAL, C4H, and 4CL, exhibited different changes (Figure 4I–III). The activities of these three enzymes exhibited a continuous increasing trend in the slight acid-treated black beans during germination. In the control treatment, the PAL activity significantly increased in the germinated black beans compared to the activity at 0 h (Figure 4I), while the activities of C4H and 4CL significantly decreased (*p* < 0.05). Meanwhile, the activities of the C4H and 4CL in the germinated black beans slightly treated with acid for 24 h were significantly higher than in the control treatment (Figure 4II,III), while the PAL activity showed no significant difference compared to the control (*p* > 0.05).

As depicted in Figure 4, the activities of three antioxidant enzymes (APX, CAT, and POD) in the germinated black beans were significantly increased (*p* < 0.05) compared to the non-germinated black beans (0 h). The activities of CAT and POD in the germinated black beans slightly treated with acid for 24 h were 5.89 times and 8.54 times higher, respectively, than those at 0 h (Figure 4V,VII). However, there was no significant difference in the activities of these three antioxidant enzymes between the control and the slight acid-treated black beans (*p* > 0.05). The activity of SOD in the slight acid-treated black beans decreased significantly during germination (Figure 4VI). These results indicate that slight acid germination treatment activates the antioxidant enzyme system in black beans.

### 3.5. Changes in Relative Gene Expression Levels of Enzymes Involved in Flavonoid Biosynthesis of Short-Germinated Black Beans

**Figure 5:**
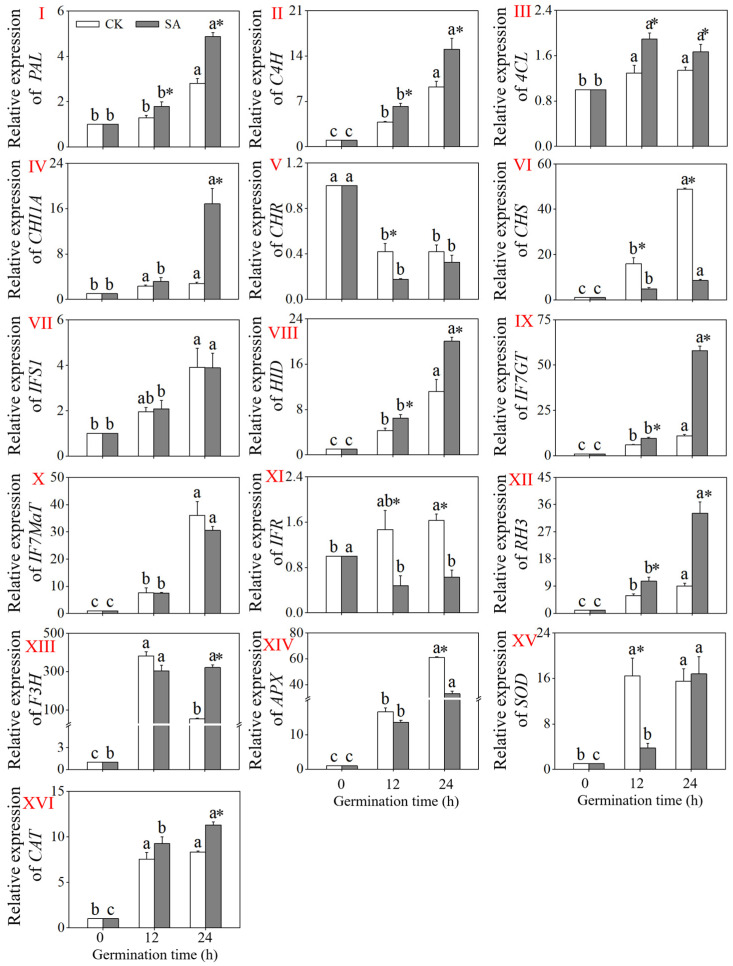
Effects of slight acid treatment in relative gene expression of *PAL* (**I**), *C4H* (**II**), *4CL* (**III**), *CHI1A* (**IV**), *CHR* (**V**), *CHS* (**VI**), *IFS* (**VII**), *HID* (**VIII**), *IF7GT* (**IX**), *IF7MaT* (**X**), *IFR* (**XI**), *RH3* (**XII**), *F3H* (**XIII**), *APX* (**XIV**), *SOD* (**XV**), and *CAT* (**XVI**) in black soybean sprouts during germination. The gene expression of actin (reference gene) was set to 1. Different lowercase letters indicate significant differences among the germination time under the same treatment (*p* < 0.05). * Indicates a significant difference between the SA treatments and CK at the same germination time (*p* < 0.05). SA: slight acid treatment. CK: water treatment.

The changes in the expression levels of genes associated with the phenylpropane metabolic pathway and antioxidant system are depicted in Figure 5. Compared to non-germinated black soybeans, the expression levels of *PAL*, *C4H*, *4CL*, *CHI1A*, *CHS*, *IFS*, *HID*, *IF7GT*, *IF7MaT*, *RH*3, and *F3H* in the black beans after 24 h of germination significantly increased (*p* < 0.05). Furthermore, after 24 h of germination, the expression levels of *PAL*, *C4H*, *CHI1A*, *HID*, *IF7GT*, *RH*3, and *F3H* in the black soybean sprouts slightly treated with acid were significantly higher than those in the control treatment (*p* < 0.05), while the expression levels of *CHS* and *IFR* were significantly lower than in the control treatment (*p* < 0.05). There was no significant change in the expression of other genes in the phenylpropane metabolic pathway between the two treatments (*p* > 0.05). In the antioxidant system, the gene expression in the black soybeans slightly treated with acid continued to increase with the germination time. Compared to the control treatment, the expression level of *CAT* in the black beans after 24 h of germination was significantly increased (*p* < 0.05), while *APX* was significantly decreased, and the expression level of *SOD* showed no significant change (*p* > 0.05).

### 3.6. Correlation between Indicators of Short-Germinated Black Soybeans under Slight Acid Treatment

**Figure 6:**
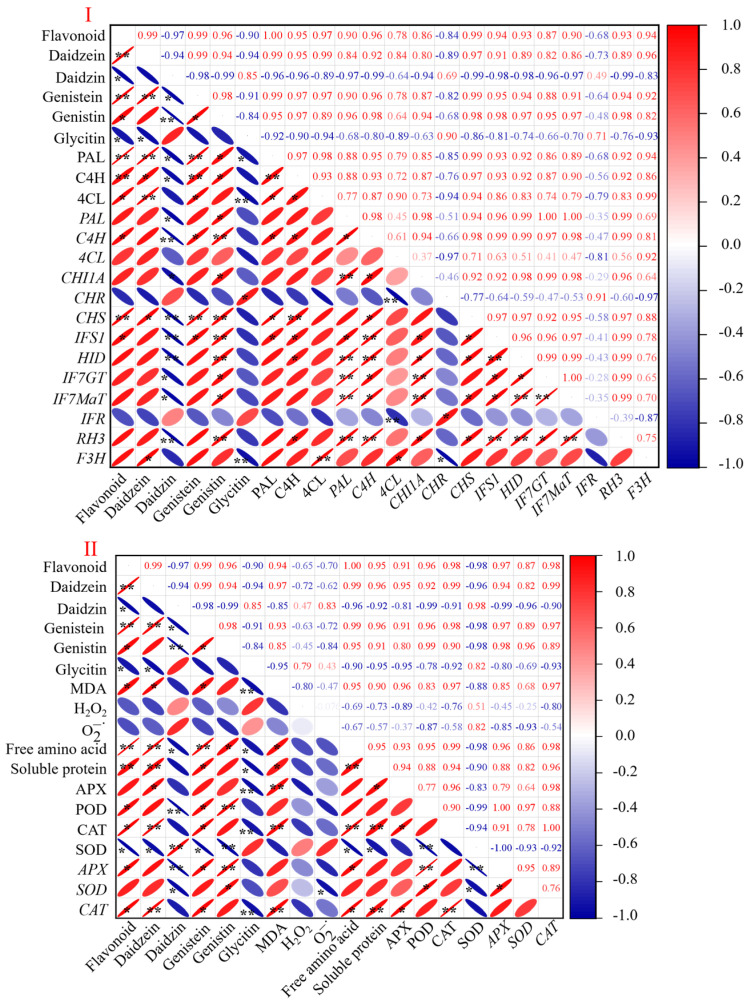
Correlation analysis of flavonoids and their monomers with indicators of flavonoid synthesis metabolism (**I**) and other indicators (**II**) in short-germinated black beans under slight acid treatment during germination. The negative and positive correlations are represented by blue and red hues, respectively. The correlation coefficients cover the spectrum from −1 to 1. * and ** indicate that the correlation coefficient is significant at the p-value levels of 0.05 and 0.01, respectively.

The Pearson correlation analysis of the enzyme activity and gene expression in the flavonoid and monomer synthesis, as well as the antioxidant system in the short-germinated black beans under a slight acid treatment, is depicted in Figure 6. The purpose of this analysis is to gain a clearer understanding of the correlation between the flavonoid content in the sprouts and other indicators. Figure 6I illustrates a significant positive correlation between the total flavonoid content and the daidzein (*p* < 0.01), genistein (*p* < 0.01), and genistin contents (*p* < 0.05), while it also shows a significant negative correlation with the glycitin and daidzin contents (*p* < 0.05). Meanwhile, the flavonoid content is significantly positively correlated with the activity of three flavonoid synthesis enzymes (PAL, C4H, and 4CL) and the gene expression of *C4H*, *CHS*, and *IFS*1 but not significantly correlated with the gene expression of other flavonoid synthesis metabolic enzymes (*p* > 0.05). Furthermore, there are significant differences in the correlation between each individual flavonoid and its related gene expression. The concentration of daidzin is significantly positively correlated with the gene expression levels of *C4H*, *CHS*, *HID*, and *IFS*1 (*p* < 0.01), while the concentration of genistin is significantly negatively correlated with these gene levels (*p* < 0.01). Furthermore, the flavonoid content is significantly and positively correlated with the levels of free amino acids, soluble proteins, and MDA (*p* < 0.05). The content of free amino acids and soluble proteins is negatively correlated with the enzyme activity and gene expression in the antioxidant system, except for the SOD activity (Figure 6II).

## 4. Discussion

Flavonoids are secondary metabolites produced during plant development through phenylpropane metabolism, and they have estrogen-like properties. The stage of seed germination is critical for determining metabolic pathways. Endogenous enzymes are activated during germination, leading to the breakdown of macromolecules, such as proteins, carbohydrates, and lipids, into smaller molecules [25]. Some bioactive compounds, such as isoflavones [26], phenolic compounds [27], and γ-aminobutyric acid [5], are significantly increased after germination. In this study, we found that the flavonoid content of black soybeans initially increased and then declined as the germination time, pH, and treatment solution concentration increased. Under ideal conditions using the response surface technique, the highest flavonoid content of the short-germinated black soybeans could reach 2.32 mg/g FW.

The two primary chemical components of soybean flavonoids are aglycones (mostly daidzein, glycitein, and genistein) and glycosides (primarily daidzin, glycitin, and genistin). Aglycones outperform isoflavone glycosides in terms of antioxidant activity [28]. The levels of daidzein and genistein significantly increased under the slight acid treatment, suggesting that aglycones played a role in the resistance to the acid during the growth of the black soybean sprouts. The substantial rise in daidzein, genistein, glycitin, and genistin was in line with the increase in flavonoid content. The gene *HID* regulates the production of daidzein and genistein, while the gene *IF7GT* converts them into daidzin and genistin, respectively [29]. Under the slight acid treatment, the relative expression of *HID* in the 24 h black soybean sprouts was significantly upregulated, which was consistent with the increase in the daidzein and genistein contents. The upregulation of *IF7GT* did not lead to an increase in the daidzin and genistin contents. Additionally, the daidzin content in the black soybean sprouts decreased gradually with the increase in the germination time. This may be attributed to differences in the regulatory mechanisms between gene transcription and daidzin synthesis proteins or to the transformation of daidzin into other substances. The content of glycitein in the black soybeans was not determined; it may be too low.

Exogenous stimulation is considered a simple and effective technique for enhancing the production of secondary metabolites in plants. The accumulation of numerous secondary metabolites has been shown to be a defensive response to environmental stimuli [30]. In plants, the pH can affect seed germination, leading to different levels of stress and ultimately increasing the contents of secondary metabolites [31]. The pH of the naturally slightly alkaline cytoplasm of plant cells may decrease during glycolysis when treated with exogenous acid [32]. A change in the cytosolic pH can significantly impact the metabolism and lead to cell damage [33]. In the current investigation, the growth of the black soybean sprouts was significantly inhibited under acidic treatment (Figure 3I). Additionally, the substances indicating the degree of damage (the content of MAD, $O_{2}^{-.}$, and H_2_O_2_) increased significantly, suggesting that the slight acid treatment had a stress-inducing effect on the black soybean sprouts. This may have altered the cytoplasmic pH, leading to a further increase in flavonoids in the black soybean sprouts during germination under acidic treatment.

The levels of MDA, $O_{2}^{-.}$, and H_2_O_2_ decreased significantly under the CK treatment with the increase in the germination time, as germination broke the seeds’ dormancy state. The flavonoids in the black soybeans during germination were highly positively correlated with MDA while being negatively correlated with H_2_O_2_ and $O_{2}^{-.}$. This may be due to the relieving effect of H_2_O_2_ and $O_{2}^{-.}$ brought about by germination being greater than the stress effect brought about by the slight acid treatment. Plants have developed an antioxidant defense mechanism comprising both enzyme and non-enzyme components to mitigate excessive inhibition [34]. As a result of acid stress, the free amino acid content of the black soybean sprouts increased significantly (Figure 3VI), as did the activity of the antioxidant enzyme APX (Figure 4IV). Under the slight acid treatment, the activity of SOD steadily decreased as the germination time increased. This might be related to the generation of reactive oxygen species beyond the clearance range of SOD. Furthermore, the correlation analysis indicates a positive correlation between flavonoids and free amino acids, soluble proteins, APX, POD, and CAT, suggesting a response of the black soybeans to environmental stress. The metabolic core process in the production of flavonoids involves three enzymes: PAL, C4H, and 4CL [22]. In our study, the activities of PAL, C4H, and 4CL increased significantly with the duration of germination, corresponding to the finding that the flavonoid content of the black soybean sprouts also increased with the germination time. These results indicate that the slight acid treatment promoted the accumulation of flavonoids in the black soybean sprouts by increasing the PAL, C4H, and 4CL activities. The strong positive correlation between the flavonoids and PAL, C4H, and 4CL further supports the previous finding.

The analysis of relative gene expression variations can elucidate the mechanism behind flavonoid enrichment through a slight acid treatment at the gene level. Most genes associated with the flavonoid biosynthesis pathway (including *PAL*, *4CL*, *CHI1A*, *CHR*, *HID*, *IF7GT*, *IFMaT*, *RH3*, and *F3H*) were significantly upregulated in the 24 h black soybean sprouts compared to the CK. This demonstrates that gene expression in the flavonoid production pathway varies under acidic conditions. The relative expressions of *PAL* and *4CL* in the black soybean sprouts were significantly upregulated by the slight acid treatment, which was consistent with the increased enzyme activity and the accumulation of flavonoids in the black soybean sprouts enhanced by a slight acid treatment. IFS and CHR are key enzymes in the flavonoid metabolic pathways of the phenylpropane metabolic route, and they are essential factors that influence flavonoid production [35]. In this study, the relative expression of *IFS1* was dramatically upregulated in the black soybean sprouts under a slight acid treatment, while *CHR* was notably downregulated. The relative expression of *CAT* was dramatically increased. The increases in the APX and C4H activities, however, were not consistent with their respective gene expression levels during germination, which could be due to spatial and temporal variations in gene expression under experimental conditions [22].

In conclusion, RSM was employed in this study to optimize the germination conditions of black soybeans. The germination duration was 24 h, the concentration was 126.20 mM, and the pH was 5.10. Furthermore, the morphology, physiology, antioxidant system, key enzymes, and gene expression of black soybean sprouts were studied under deionized water and a slight acid treatment. The findings reveal that the slight acid treatment inhibited the growth of the black soybean sprouts, increased the content of MDA, $O_{2}^{-.}$, and H_2_O_2_. The activities of antioxidase and flavonoid synthesis-related enzymes were increased, along with the relative expressions of their genes. This research lays the groundwork for the development and metabolic activity of black soybean sprouts, as well as the process of enriching flavonoids under a slight acid treatment.

## Figures and Tables

**Table 1:** Encoded and real levels for the germination process of black soybean sprouts and flavonoid contents of each treatment group.

Independent Variables		Levels		
		−1	0	1
X1: germination time (h)		12	24	36
X2: pH		4	5	6
X3: concentration (mM)		90	120	150
**No.**	**X1: Germination time (h)**	**X2: pH**	**X3: Concentration (mM)**	**Y: Flavonoid** **content (mg/g FW)**
1	−1	−1	0	1.66 ± 0.06
2	1	−1	0	1.72 ± 0.04
3	−1	1	0	1.84 ± 0.09
4	1	1	0	1.81 ± 0.05
5	−1	0	−1	1.79 ± 0.04
6	1	0	−1	1.79 ± 0.08
7	−1	0	1	1.86 ± 0.07
8	1	0	1	1.96 ± 0.07
9	0	−1	−1	1.75 ± 0.04
10	0	1	−1	1.87 ± 0.06
11	0	−1	1	1.86 ± 0.04
12	0	1	1	2.03 ± 0.05
13	0	0	0	2.29 ± 0.04
14	0	0	0	2.31 ± 0.07
15	0	0	0	2.29 ± 0.08
16	0	0	0	2.30 ± 0.05
17	0	0	0	2.31 ± 0.04

Note. “FW”: fresh weight.

## Data Availability

The original contributions presented in the study are included in the article/Supplementary Materials, further inquiries can be directed to the corresponding author.

## Supplementary Materials

The following supporting information can be downloaded at: https://www.mdpi.com/article/10.3390/foods13060868/s1, Figure S1. Effects of germination time (I), pH (II), and solution concentration (III) on flavonoid content. According to Tukey’s multiple comparisons, different lowercase letters indicate significant differences at *p* < 0.05. Table S1. Primer sequences used in the study. Table S2. ANOVA analysis for the response variables.
