# Supplementary material for: Enrichment of Flavonoids in Short-Germinated Black Soybeans (Glycine max L.) Induced by Slight Acid Treatment
Source: Foods. 2024 Mar 13;13(6):868. doi: 10.3390/foods13060868 (PMC10969485; doi:10.3390/foods13060868)
Supplement: Supplementary file 1 [file foods-13-00868-s001.zip › foods-2905329-supplementary.pdf]

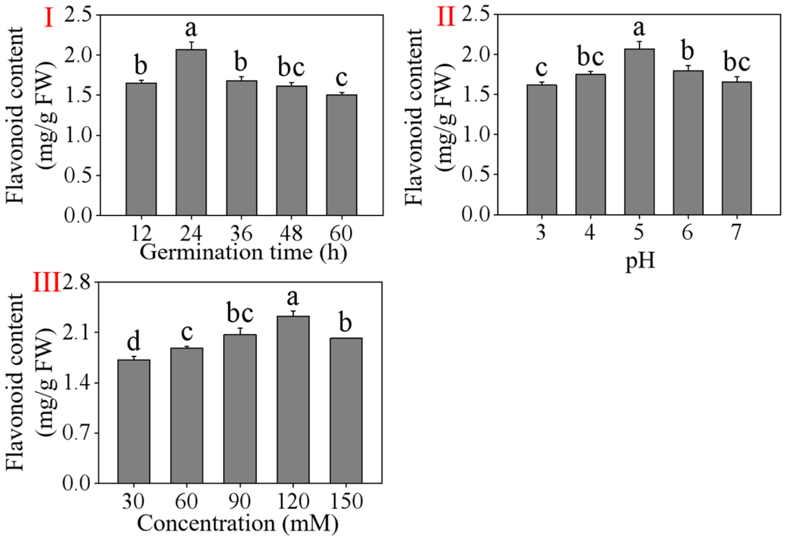

Figure S1. Effects of germination time (I), pH (II), and solution concentration (III) on flavonoid content. According to Tukey's multiple comparisons, different lowercase letters indicate significant differences at $p < 0.05$.

Table S1. Primer sequences used in the study.

Gene Name	Forward Primer (5'-3')	Reverse Primer (5'-3')
Actin	CTTCCCTCAGCACCTTCCAA	GGTCCAGCTTTCACACTCCAT
PAL	AGCAACACAACCAGGATGTCAA	CAATTGCTTGGCAAAGTGCA
C4H	AGGCGAGATCAACGAAGACAAC	GTTCACAAGCTCAGCAATGCC
4CL	AGGCAATGTACGTGGACAAGCT	TCCGAGAGGACAGAGAAGTGGA
CHI1A	GGCGCTGAATACTCAAAGAAGG	AGAGGCACCAGGTGCAAAATT
CHR	CAAAGCCATTGGAGTCAGCAA	CCATGCAAGGTTCATCTCCACT
CHS	GCTTGTTGTCTGTTCTGAG	CACCTTCACTGTCTGGAG
IFS1	AGAATTCCGTCCCGAGAGGTT	TGCCATTCCTGAAGTAGCCAA
HID	CACTCTTGCCTGCTCTAAGTT	CTCAACGGTGTGGTGGTAG
IF7GT	CCCCACCATTCACCCAAC	TGGCAAGCGTAACTCAAGG
IF7MaT	CCCTCTCTTCAAACCTCTCAG	TGGTGGCTTGTTATTCCTATCG
IFR	AGATGGAAATGTGAAAGGAGCG	TGTGCACGGCTTTGTTCAAG
RH3	CCTTGAACGTGATGTAGGCTG	GGGCACTTGTTCCTTGTTCTT
F3H	TTACCTGGCCCAGGAGAAAAC	ATTCCGGCAAGAGAAATCACTG
POD	GCTTTGAGCACCATTAGA	TTGGTGAAGGGTCTAGTA
SOD	TGGTCTCCATGGCTTCCAT	GCTAACGGTACCATCATCA
CAT	ACTACAAATTCTGGTGCTCCTA	TGCAAGCTTCTCCACAAGA

Table S2. ANOVA analysis for the response variables.

Variables	Sum of squares	df	Mean square	F value	P value
Model	0.8958	9	0.0995	592.95	< 0.0001
X_1: germination time (h)	0.0021	1	0.0021	12.59	0.0094
X_2: pH	0.0392	1	0.0392	233.53	< 0.0001
X_3: concentration (mM)	0.0335	1	0.0325	193.69	< 0.0001
X_1X_2	0.0020	1	0.0020	12.06	0.0104
X_1X_3	0.0025	1	0.0025	14.89	0.0062
X_2X_3	0.0006	1	0.0006	3.72	0.0950
X_1^2	0.3420	1	0.3420	2037.45	< 0.0001
X_2^2	0.2792	1	0.2792	1663.23	< 0.0001
X_3^2	0.1146	1	0.1146	682.91	< 0.0001
Residual	0.0012	7	0.0002		
Lack of fit	0.0008	3	0.0003	2.58	0.1908
Pure error	0.0004	4	0.0001		
Total	0.8970	16			
	R^2=0.9987	R^2_{adj}=0.997	R^2_{Pre}=0.9855	C.V.%=0.6586	
